# Associations of Increased Plant Protein Intake Ratio with Adherence of Low-Protein Diet, Acid-Base Status, and Body Composition in CKD Stage 3–5

**DOI:** 10.3390/nu17162649

**Published:** 2025-08-15

**Authors:** Bang-Gee Hsu, Li-Xia Tong, Hung-Hsiang Liou, Chih-Hsien Wang, Yu-Li Lin

**Affiliations:** 1 Division of Nephrology, Hualien Tzu Chi Hospital, Buddhist Tzu Chi Medical Foundation, Hualien 97010, Taiwan; gee.lily@msa.hinet.net (B.-G.H.); wangch33@gmail.com (C.-H.W.); 2 School of Medicine, Tzu Chi University, Hualien 97010, Taiwan; 3 Division of Dietetics and Nutrition, Hualien Tzu Chi Hospital, Buddhist Tzu Chi Medical Foundation, Hualien 97010, Taiwan; tonglixia@tzuchi.com.tw; 4 Division of Nephrology, Department of Internal Medicine, Hsin-Jen Hospital, New Taipei City 24243, Taiwan; hh258527@ms23.hinet.net

**Keywords:** plant protein intake, low-protein diet, potential renal acid load, metabolic acidosis, body composition, handgrip strength

## Abstract

**Background/Objectives**: Increasing evidence indicates that a vegetarian diet may provide renal protection and improve metabolic health in patients with chronic kidney disease (CKD). However, transitioning from an omnivorous to a vegetarian diet can be challenging. A more practical alternative could be to increase the consumption of plant protein. In this cross-sectional study, we investigated the association between increased plant protein intake and adherence to a low-protein diet (LPD) and the effect on biochemical parameters, body composition, and muscle strength in patients with non-dialysis CKD stages 3–5. **Methods**: The daily dietary intake of 377 patients, aged 68.5 ± 12.1 years, was evaluated using a quantitative food frequency questionnaire. Plant protein intake percentage was calculated as (daily plant protein intake/total protein intake) × 100%, and the potential renal acid load (PRAL) was estimated. A LPD was defined as a diet with a daily protein intake of <0.8 g/kg of body weight. Anthropometric measurements, body composition, and handgrip strength were assessed in a subgroup comprising 260 patients. The lean tissue index (LTI) and fat tissue index (FTI) were calculated by dividing lean mass and fat mass (kg) by the height in m^2^, respectively. **Results**: Of the included 377 patients, 69.5% adhered to the LPD. Further, a 10% increase in plant protein intake was associated with a 20% increase in the likelihood of LPD adherence (OR, 1.20, 95% CI, 1.06 to 1.37), lower PRAL (β = −1.10 per 10% increase, 95% CI, −1.63 to −0.57), and higher serum bicarbonate levels (β = 0.24, 95% CI, 0.02 to 0.45). Analysis of the 260-patient subgroup revealed that a 10% increase in plant protein intake was associated with lower body mass index (β = −0.82, 95% CI, −1.05 to −0.59), FTI (β = −0.71, 95% CI, −1.01 to −0.40), waist circumference (β = −2.11, 95% CI, −2.80 to −1.41), hip circumference (β = −1.25, 95% CI, −1.75 to −0.75), waist-to-hip ratio (β = −0.91, 95% CI, −1.44 to −0.38), and waist-to-height ratio (β = −1.25, 95% CI, −1.71 to −0.80). There was no significant association between increased plant protein intake and LTI and handgrip strength. **Conclusions**: Increased intake of plant protein can reduce dietary acid load, alleviate metabolic acidosis, and potentially improve adiposity parameters without compromising lean mass and handgrip strength.

## 1. Introduction

Chronic kidney disease (CKD) is a major global public health concern that affects approximately 10–12% of the global population [1,2]. In addition to its progression to end-stage renal disease, CKD significantly contributes to cardiovascular events and global mortality [3]. Therefore, slowing the decline in kidney function and addressing related metabolic complications are essential components of CKD management. Of the various therapeutic strategies, dietary modification plays a central and modifiable role in improving patient outcomes.

A vegetarian diet that primarily comprises plant-based food products has been reported to have substantial health benefits, including in the prevention of cardiovascular disease, hypertension, diabetes mellitus (DM), dyslipidemia, and certain cancers, in the general population [4,5]. In patients with CKD, a vegetarian diet has been reported to decrease serum levels of phosphorus and fibroblast growth factor-23 [6] and significantly reduce levels of protein-bound uremic toxins such as p-cresyl sulfate and indoxyl sulfate [7]. Furthermore, a landmark study demonstrated that a supplemented vegetarian very low-protein diet (sVLPD) significantly slows renal disease progression, alleviates metabolic acidosis, and improves calcium–phosphorus balance compared with a conventional low-protein diet (LPD) [8]. Despite these benefits, most patients with CKD following an omnivorous diet find it challenging to switch to a fully vegetarian or vegan diet. Therefore, increasing the proportion of plant protein in the diet has been recently proposed as a more practical alternative dietary strategy for this population [9]. However, the benefits of increasing plant protein intake in the diet of patients with CKD remain to be fully elucidated.

This cross-sectional study aimed to explore the associations between increased plant protein intake and adherence to LPD and the effect on metabolic parameters, body composition, and muscle strength in patients with non-dialysis CKD stages 3–5.

## 2. Materials and Methods

### 2.1. Study Design and Participants

This cross-sectional study was conducted at the outpatient nephrology clinic of Hualien Tzu Chi Hospital in eastern Taiwan. Patient enrollment and data collection were conducted between September 2022 and January 2024. CKD stages 3–5 were defined as an estimated glomerular filtration rate (eGFR) of <60 mL/min/1.73 m^2^ for at least 3 months. Patients with CKD stages 3–5 and aged > 20 years were invited to participate. All patients were followed-up every 1–3 months for multidisciplinary renal care and underwent dietary counseling on adherence to a CKD-specific diet. The recommended daily intake was 25–35 kcal/kg of body weight for energy and 0.6–0.8 g/kg for protein, which is based on the 2020 update of the Kidney Disease Outcomes Quality Initiative Clinical Practice Guideline for Nutrition in CKD [10]. The exclusion criteria were the presence of active infection or acute myocardial infarction within the past 3 months; dialysis treatment; history of stroke, pacemaker implantation, or limb amputation; apparent uremic symptoms or signs; being wheelchair-bound or bedridden; and refusal to participate.

Data on basic demographics; comorbidities including DM, hypertension, and hyperlipidemia; and medications including statins, diuretics (thiazide and furosemide), insulin, sodium bicarbonate, potassium binders, and phosphate binders were obtained from patients’ electronic medical records. The following laboratory data that were routinely monitored during outpatient visits were retrieved for analysis: levels of serum creatinine, potassium, total calcium, phosphorus, albumin (bromocresol green method), venous bicarbonate, fasting glucose, total cholesterol, and triglyceride, as well as eGFR, calculated using the Modification of Diet in Renal Disease equation and the urine protein-to-creatinine ratio.

This study was approved by the Institutional Review Board of Hualien Tzu Chi Hospital (IRB No. 111-184-B); written informed consent was obtained from all patients.

### 2.2. Assessment of Dietary Intake and Nutrient Determination

A validated quantitative food frequency questionnaire (FFQ) comprising 64 items was used to assess patients’ dietary intake [11]. The listed food items were organized into the following key categories: fish, seafood, and meat; eggs and dairy products; soy-based and other plant-derived protein sources; vegetables; fruits and nuts; staple foods such as rice, noodles, bread, and oats; beverages; and cooking oils. For portion-size education, each food item was accompanied by an average of five portion-size photographs from the Taiwan Department of Health. Included patients were interviewed by an investigator who was trained by a dietitian on reporting consumption frequency (daily, weekly, monthly, or never) and comparing the patients’ portion sizes with the photographs (similar, half, or double). Nutrient intake was calculated based on the reference values from the Taiwan’s Food Composition Database. A detailed description of the FFQ has been previously reported [11]. Among 398 patients with CKD who completed the FFQ, those with a daily energy intake of >4000 kcal (n = 2) or <400 kcal (n = 19) were excluded owing to invalid assessments. Finally, 377 patients with CKD stages 3–5 who completed the FFQ were included in the final analysis.

Plant protein intake percentage was calculated as (daily plant protein intake/total protein intake) × 100%. Potential renal acid load (PRAL) was calculated as 0.49 × total protein intake (in g/day) + 0.037 × phosphorus intake (in mg/day) − 0.021 × potassium intake (in mg/day) − 0.026 × magnesium intake (in mg/day) − 0.013 × calcium intake (in mg/day) [12].

### 2.3. Assessment of Anthropometric Data, Body Composition, and Handgrip Strength

Height and weight were measured with the patients standing barefoot and wearing light clothing; body mass index (BMI) was subsequently calculated. Waist circumference was measured at the midpoint between the lower edge of the last rib and the top of the iliac crest, whereas hip circumference was measured at the widest part of the hips and buttocks. The association between plant protein intake and anthropometric measurements, body composition, and handgrip strength was further explored in a subgroup of 260 patients. Lean mass and fat tissue mass were assessed using a portable whole-body multifrequency bioimpedance spectroscopy device (BCM, Fresenius Medical Care, Bad Homburg, Germany) that takes hydration status into account and has been widely used to evaluate body composition in patients with CKD [13]. The lean tissue index (LTI) was calculated using the formula lean mass (in kg)/height (in m^2^), whereas the fat tissue index (FTI) was calculated using the formula fat mass (in kg)/height (in m^2^). Handgrip strength was assessed by asking the patients to grip a dynamometer (Jamar Plus Digital Hand Dynamometer, SI Instruments Pty Ltd., Hilton, Australia) while standing with their arm bent at a right angle and their elbow close to the body. Measurements were taken thrice at 1 min rest intervals, and the mean value was used for analysis [14].

### 2.4. Statistical Analyses

Continuous variables are expressed as mean ± standard deviation or median (interquartile range, IQR), depending on the data distribution on the Kolmogorov–Smirnov test. Categorical variables are presented as absolute numbers (n) and corresponding percentages (%). The association between increased plant protein intake and adherence to LPD was assessed using univariate logistic regression. The association between increased plant protein intake and metabolic and biochemical parameters and body composition was evaluated using multivariate linear regression while adjusting for age; sex; DM; hypertension; hyperlipidemia; eGFR; daily energy intake and protein intake; and use of statins, diuretics, sodium bicarbonate, potassium binders, and calcium carbonate. All data were analyzed using SPSS for Windows (version 19.0, IBM Corp., Armonk, NY, USA). A *p* value of <0.05 was considered statistically significant.

## 3. Results

The baseline characteristics of the included 377 patients are presented in Table 1. The mean age was 68.5 ± 12.1 years, with 42.2% comprising women. Further, 48.0% of the patients were in stage 3, 32.6% in stage 4, and 19.4% in stage 5. The prevalences of DM, hypertension, and hyperlipidemia were 60.2%, 77.7%, and 75.1%, respectively. The median eGFR was 28.9 mL/min/1.73 m^2^, and the median serum potassium and phosphorus levels were 4.4 mmol/L and 3.6 mg/dL, respectively.

Table 2 summarizes the dietary intake of macro- and micronutrients in this study population. Most patients (94.4%) followed an omnivorous diet. The mean daily energy intake was 1272 ± 590 kcal, and total protein intake was 45 ± 22 g/day. The median dietary fiber intake was 11.1 g/day (IQR, 7.8–15.8 g). Adherence to LPD was observed in 69.5%, and the mean plant protein percentage was 56 ± 18%.

The association between plant protein intake and the likelihood of LPD adherence is presented in Table 3. Each 10% increase in plant protein intake was associated with 1.20-fold higher odds of LPD adherence (95% CI, 1.06 to 1.37, *p* = 0.005). Compared with patients whose plant protein intake proportion was ≤50%, those with a plant protein intake proportion of >50% had 1.59-fold higher odds of LPD adherence (95% CI, 1.02 to 2.47, *p* = 0.041).

Table 4 presents the associations between plant protein intake proportion and metabolic parameters. Increased plant protein intake was associated with lower PRAL (β = −1.10 per 10% increase, 95% CI, −1.63 to −0.57, *p* < 0.001) and higher serum bicarbonate levels (β = 0.24 per 10% increase, 95% CI, 0.02 to 0.45, *p* = 0.031) but was not significantly associated with serum glucose, total cholesterol, triglyceride, potassium, calcium, or phosphorus levels. Further analyses stratified by CKD stages 3 and 4–5 and by DM status are provided in Tables S1 and S2. Notably, a higher proportion of plant protein intake was significantly associated with lower serum phosphorus levels in the non-DM group (β = −0.08 per 10% increase, 95% CI, −0.14 to −0.02, *p* = 0.014), but no such association was observed in DM patients (*p* for interaction = 0.026).

Subgroup analysis of 260 patients revealed that a 10% increase in plant protein intake was significantly associated with lower BMI (β = −0.82, 95% CI, −1.05 to −0.59, *p* < 0.001), lower FTI (β = −0.71, 95% CI, −1.01 to −0.40, *p* < 0.001), reduced waist circumference (β = −2.11, 95% CI, −2.80 to −1.41, *p* < 0.001), reduced hip circumference (β = −1.25, 95% CI, −1.75 to −0.75, *p* < 0.001), lower waist-to-hip ratio (β = −0.91, 95% CI, −1.44 to −0.38, *p* = 0.001), and lower waist-to-height ratio (β = −1.25, 95% CI, −1.71 to −0.80, *p* < 0.001). In contrast, increased plant protein intake was not significantly associated with LTI and handgrip strength (Table 5).

## 4. Discussion

The salient finding of this cross-sectional study on patients with non-dialysis CKD stages 3–5 was that increased plant protein intake is associated with greater adherence to LPD, lower dietary acid load, alleviation of metabolic acidosis, and a potential decrease in adiposity. Importantly, these benefits were achieved without compromising on LTI and handgrip strength.

Glomerular hyperfiltration and increased intraglomerular pressure are pivotal in CKD progression [15,16]. A protein-restricted diet can decrease intraglomerular pressure through the vasoconstriction of afferent arterioles and modulation of the angiotensin pathway [17]. Compelling evidence from clinical trials has demonstrated that LPD or sVLPD slows CKD progression [8,18,19]. Unfortunately, in a real-world setting, adherence to LPD is challenging for patients with CKD, with globally reported adherence rates ranging from 34.5% to 54.0% [20,21,22,23]. Our study indicated that increasing plant protein intake can be a practical approach to allow adherence to LPD. Each 10% increase in plant protein intake was associated with 20% higher odds of adhering to LPD, especially in those whose diet included a plant protein proportion of >50%. Patients following a vegetarian diet had nearly twice the odds of LPD adherence compared to those following an omnivorous diet, although this association was not significant, likely because of the limited number of vegetarians in the study population. Our findings are in accordance with those of a clinical trial by Garneata et al., which revealed that a very low-protein diet (0.3 g/kg/day) could be successfully implemented by following a vegetarian diet [8].

Metabolic acidosis progresses as renal function declines and can adversely impact bone mineralization, skeletal muscle homeostasis, and insulin sensitivity while accelerating renal deterioration [24,25]. Our study findings suggest that increased plant protein intake mitigates metabolic acidosis, primarily through reduced renal acid load. This effect is mainly attributed to the increased consumption of fruits and vegetables, which are rich in bicarbonate precursors (citrate and malate) and the decreased consumption of animal proteins that typically contain sulfur-containing amino acids [26]. Similarly, increased plant protein intake was reported to be associated with higher serum bicarbonate levels in the Chronic Renal Insufficiency Cohort (CRIC), a large CKD study including patients with eGFR of 20–70 mL/min [27].

The subgroup analysis in our study revealed a prominent association between increased plant protein intake and reduced adiposity, including BMI, waist and hip circumference, and waist-to-hip and waist-to-height ratios. In line with the findings of the present study, the Rotterdam study, a large-scale prospective cohort study on the general middle-aged and elderly population in the Netherlands, confirmed that higher adherence to a plant-based diet contributed to reduced BMI, waist circumference, fat mass index, and body fat percentage over a median follow-up period of 7 years [28]. It is reasonable to speculate that among patients with CKD and central obesity, modification of adiposity parameters by increasing plant protein intake may mitigate the adverse consequences of central obesity, such as insulin resistance, inflammation, vascular dysfunction, and CKD progression [29,30]. In this study, the absence of associations between increased plant protein intake and lower LTI, handgrip strength, and serum albumin levels indicated that increasing the plant protein intake as a part of individualized dietary strategies under regular dietitian supervision would not compromise overall nutritional status and skeletal muscle health.

Although phosphate from plant-based protein, which exists in the form of phytate, is well-known to have a lower gastrointestinal bioavailability compared with animal protein [31], our main results did not show any association between increased plant protein intake and lower serum phosphorus levels and calcium–phosphorus products. This may be explained by the fact that most patients were in CKD stages 3 and 4, during which renal phosphorus excretion remains relatively preserved. This was further supported by the low median serum phosphorus levels and the limited use of phosphate binders in our cohort. Consistent with our findings, the CRIC we previously mentioned did not report any significant association between plant protein intake and serum phosphate levels [27]. However, they found that a higher plant protein intake was associated with lower serum levels of FGF-23, a biomarker that reflects chronic kidney disease–mineral and bone disorder, earlier than serum phosphate.

Interestingly, in subgroup analyses, we observed that the potential phosphorus-lowering effect of higher plant protein intake may be more pronounced in non-diabetic CKD patients. Given the limited evidence directly comparing diabetic and non-diabetic CKD populations in this context, further studies are warranted to confirm and clarify these differences.

This real-world study explored the potential benefits of increased plant protein intake in patients with CKD stages 3–5. A major strength of this study was the simultaneous examination of the association between increased plant protein intake and various clinical and metabolic parameters. However, several limitations should be acknowledged. First, this study used a face-to-face questionnaire approach; therefore, the higher adherence to LPD compared with that in previous studies may be partially because of protein intake being underreported. More objective methods for assessing daily protein intake, such as the Maroni formula based on 24 h urinary urea excretion, were unavailable. Second, the estimated energy intake was low in this cohort. Although FFQ is a widely used method in nutritional epidemiology studies, its accuracy in assessing dietary intake adequacy may be hampered by recall bias, portion-size misreporting, or a limited number of food items included. Nevertheless, inadequate energy intake is common in a CKD population [32,33] which highlights the importance of paying special attention to maintaining sufficient energy intake in this group. Third, the number of participants following a vegan or lacto-ovo vegetarian diet was limited; this precluded direct comparisons between vegetarian and non-vegetarian diets. This limitation may account for the lack of a significant association between a vegetarian diet and LPD adherence despite the large effect size. However, because our data reflected a common real-world scenario, we analyzed plant protein intake as a percentage of total protein intake rather than categorizing participants based on dietary patterns. Fourth, owing to clinical feasibility in an outpatient setting, we used venous rather than arterial blood samples for acidosis assessment. Nevertheless, the difference in bicarbonate levels between arterial and venous blood gases is minimal [34]. Fifth, some confounding factors, such as physical activity, which may affect anthropometric data and body composition, were unavailable in this study. Sixth, the cross-sectional design cannot establish causality, and the limited number of patients with CKD stage 5 restricts the generalizability of our findings to this stage. Finally, our findings should be extrapolated and applied with caution to other racial and ethnic populations because dietary habits can differ widely.

## 5. Conclusions

Given that complete elimination of animal protein may not be feasible for many patients with CKD in a real-world setting, our findings have important clinical implications. Increasing plant protein intake can support LPD adherence and may help reduce renal acid load, alleviate metabolic acidosis, and decrease adiposity without compromising skeletal muscle mass and strength. Further large-scale studies are warranted to validate these findings.

## Figures and Tables

**Table 1:** Clinical characteristics of the study population.

Characteristics	Number of Patients (N = 377)
**Basic demographics**	
Age (years)	68.5 ± 12.1
Sex (female), n (%)	159 (42.2)
CKD stage 3, n (%)	181 (48.0)
CKD stage 4, n (%)	123 (32.6)
CKD stage 5, n (%)	73 (19.4)
**Diseases, n (%)**	
DM	227 (60.2)
Hypertension	293 (77.7)
Hyperlipidemia	283 (75.1)
**Basic measurement**	
Height (cm)	160.6 ± 8.1
Weight (kg)	67.5 ± 13.1
BMI (kg/m^2^)	26.1 ± 4.3
BMI < 18.5 kg/m^2^, n (%)	4 (1.1)
**Body composition ^a^**	
LTI (kg/m^2^)	14.8 ± 3.7
FTI (kg/m^2^)	11.3 ± 5.0
Waist circumference (cm)	90.4 ± 11.3
Hip circumference (cm)	97.5 ± 8.2
Waist-to-hip ratio (%)	92.5 ± 8.0
Waist-to-height ratio (%)	56.3 ± 7.0
**Handgrip strength ^a^**	
Right hand (kg)	24.2 ± 9.2
Left hand (kg)	22.7 ± 9.0
Average (kg)	23.5 ± 8.9
**Laboratory data**	
Creatinine (mg/dL)	2.2 (1.6–3.2)
eGFR (mL/min/1.73 m^2^)	28.9 (17.8–40.4)
Albumin (g/dL)	4.1 (3.9–4.3)
Albumin < 3.8 g/dL, n (%)	65 (17.2)
UPCR (g/g)	0.59 (0.22–1.84)
HCO3 (mmol/L)	23.1 ± 3.9
Glucose (mg/dL)	105 (94–130)
Total cholesterol (mg/dL)	146 (125–174)
Triglyceride (mg/dL)	121 (91–169)
Potassium (mmol/L)	4.4 (4.0–4.7)
Total calcium (mg/dL)	9.2 (8.9–9.5)
Phosphorus (mg/dL)	3.6 (3.3–4.2)
**Medications, n (%)**	
Statins	167(44.3)
Diuretics	111 (29.4)
Insulin	64 (17.0)
Sodium bicarbonate	50 (13.3)
Potassium binders	30 (8.0)
Phosphate binders	8 (2.1)

^a^ n = 260; CKD, chronic kidney disease; DM, diabetes mellitus; BMI, body mass index; LTI, lean tissue index; FTI, fat tissue index; eGFR, estimated glomerular filtration rate; UPCR, urine protein-to-creatinine ratio; HCO3, bicarbonate.

**Table 2:** Results of the dietary assessment through the food frequency questionnaire.

Characteristics	Number of Patients (N = 377)
**Dietary pattern, n (%)**	
Omnivorous	356 (94.4)
Lacto-ovo vegetarian	14 (3.7)
Vegan	7 (1.9)
**Nutrients**	
Carbohydrate (g/day)	156 (112–217)
Fat (g/day)	39 (25–59)
Energy intake (kcal/day)	1272 ± 590
Dietary fiber (g/day)	11.1 (7.8–15.8)
Total protein intake (g/day)	45 ± 22
DPI < 0.8 g/kg/day, n (%)	262 (69.5)
**Protein sources**	
Animal protein (g/day)	17.8 (10.0–27.3)
Plant protein (g/day)	21.7 (15.2–31.3)
Plant protein ratio (%)	56 ± 18
**Mineral intake (mg/day)**	
Potassium	1425 (994–1828)
Calcium	288 (185–381)
Magnesium	137 (97–190)
Phosphorus	537 (394–767)
**PRAL (mEq/day)**	5.6 (−0.2–12.3)

DPI, daily protein intake; PRAL, potential renal acid load.

**Table 3:** Association between increased plant protein intake and adherence to a low-protein diet (<0.8 g/kg/day) in the study population.

Protein Intake Pattern	Univariate		*p* Value
	OR	95% CI	
Per 10% increase in plant protein intake	1.20	1.06–1.37	0.005 *
Plant protein intake proportion >50%	1.59	1.02–2.47	0.041 *
Vegetarian diet	1.93	0.63–5.86	0.248

Vegetarian diet included lacto-ovo vegetarian and vegan diets. * *p* < 0.05 was considered significant.

**Table 4:** Associations between increased plant protein intake and potential renal acid load, metabolic acidosis, and metabolic parameters in the study population.

Variables	Plant Protein Intake (Per 10% Increase)		
	β	95% CI	*p*
PRAL (mEq/day)	−1.10	−1.63, −0.57	<0.001 *
HCO3 (mmol/L)	0.24	0.02, 0.45	0.031 *
Albumin (g/dL)	0.01	−0.01, 0.03	0.415
Glucose (mg/dL)	−0.83	−3.38, 1.71	0.521
Total cholesterol (mg/dL)	0.06	−2.31, 2.43	0.962
Triglyceride (mg/dL)	−3.53	−10.06, 3.00	0.289
Potassium (mmol/L)	−0.02	−0.05, 0.01	0.195
Total calcium (mg/dL)	−0.01	−0.03, 0.02	0.731
Phosphorus (mg/dL)	−0.02	−0.06, 0.02	0.328
Ca × P (mg^2^/dL^2^)	−0.20	−0.61, 0.21	0.328

Multivariate linear regression analysis was conducted after adjusting for age; sex; DM; hypertension; hyperlipidemia; eGFR; daily energy intake and protein intake; and use of statins, diuretics, sodium bicarbonate, potassium binders, and calcium carbonate. PRAL, potential renal acid load; HCO3, bicarbonate; Ca × P, calcium–phosphorus product. * *p* < 0.05 was considered significant.

**Table 5:** Associations between increased plant protein intake and anthropometric data, body composition, and handgrip strength in the subgroup.

Variables	Plant Protein Intake (Per 10% Increase)		
	β	95% CI	*p* Value
BMI (kg/m^2^)	−0.82	−1.05, −0.59	<0.001 *
LTI (kg/m^2^)	−0.03	−0.29, 0.23	0.819
FTI (kg/m^2^)	−0.71	−1.01, −0.40	<0.001 *
Waist circumference (cm)	−2.11	−2.80, −1.41	<0.001 *
Hip circumference (cm)	−1.25	−1.75, −0.75	<0.001 *
Waist-to-hip ratio (%)	−0.91	−1.44, −0.38	0.001 *
Waist-to-height ratio (%)	−1.25	−1.71, −0.80	<0.001 *
Right handgrip strength (kg)	−0.23	−0.72, 0.27	0.375
Left handgrip strength (kg)	−0.04	−0.53, 0.45	0.879
Average handgrip strength (kg)	−0.15	−0.62, 0.33	0.546

Multivariate linear regression analysis was adopted, adjusting for age; sex; DM; hypertension; hyperlipidemia; eGFR; daily energy intake and protein intake; and use of statins, diuretics, sodium bicarbonate, potassium binders, and calcium carbonate. BMI, body mass index; LTI, lean tissue index; FTI, fat tissue index. * *p* < 0.05 was considered significant.

## Data Availability

The data presented in this study are available on request from the corresponding author. The data are not publicly available due to privacy or ethical restrictions.

## Supplementary Materials

The following supporting information can be downloaded at: https://www.mdpi.com/article/10.3390/nu17162649/s1, Table S1: Associations between increased plant protein intake and potential renal acid load, metabolic acidosis, and metabolic parameters, stratified by CKD stages; Table S2: Associations between increased plant protein intake and potential renal acid load, metabolic acidosis, and metabolic parameters, stratified by DM status.
