# Supplementary material for: Associations of Increased Plant Protein Intake Ratio with Adherence of Low-Protein Diet, Acid-Base Status, and Body Composition in CKD Stage 3–5
Source: Nutrients. 2025 Aug 15;17(16):2649. doi: 10.3390/nu17162649 (PMC12389221; doi:10.3390/nu17162649)
Supplement: Supplementary file 1 [file nutrients-17-02649-s001.zip › nutrients-3795165-supplementary.pdf]

Table S1. Associations between increased plant protein intake and potential renal acid load, metabolic acidosis, and metabolic parameters, stratified by CKD stages

Variables	Plant protein intake (per 10% increase)					
	CKD stage 3 (n = 181)			CKD stage 4–5 (n = 196)		
	β	95% CI	*p*	β	95% CI	*p*
PRAL (mEq/day)	−1.32	−2.19, −0.45	0.003*	−1.00	−1.69, −0.30	0.006*
HCO3 (mmol/L)	0.35	−0.01, 0.71	0.051	0.18	−0.09, 0.46	0.195
Albumin (g/dL)	0.01	−0.02, 0.04	0.556	0.01	−0.03, 0.04	0.706
Glucose (mg/dL)	−0.46	−3.36, 2.43	0.752	−1.36	−5.56, 2.83	0.523
Total cholesterol (mg/dL)	1.50	−2.21, 5.22	0.426	−0.89	−4.20, 2.42	0.596
Triglyceride (mg/dL)	−2.42	−15.06, 10.22	0.706	−3.88	−10.46, 2.69	0.245
Potassium (mmol/L)	−0.02	−0.06, 0.03	0.481	−0.03	−0.08, 0.02	0.220
Total calcium (mg/dL)	−0.02	−0.03, 0.06	0.506	−0.01	−0.06, 0.04	0.722
Phosphorus (mg/dL)	0.01	−0.05, 0.05	0.926	−0.05	−0.11, 0.02	0.174
Ca × P (mg^2/dL^2)	0.05	−0.44, 0.55	0.835	−0.42	−1.05, 0.22	0.199

Multivariate linear regression analysis was conducted after adjusting for age; sex; DM; hypertension; hyperlipidemia; eGFR; daily energy intake and protein intake; and use of statins, diuretics, sodium bicarbonate, potassium binders, and calcium carbonate.

CKD, chronic kidney disease; PRAL, potential renal acid load; HCO3, bicarbonate; Ca × P, calcium–phosphorus product.

*$p < 0.05$ was considered significant.

Table S2. Associations between increased plant protein intake and potential renal acid load, metabolic acidosis, and metabolic parameters, stratified by DM status

Variables	Plant protein intake (per 10% increase)					
	Non-DM (n = 150)			DM (n = 227)		
	β	95% CI	*p*	β	95% CI	*p*
PRAL (mEq/day)	−1.65	−2.51, −0.79	< 0.001*	−0.62	−1.28, 0.04	0.067
HCO3 (mmol/L)	0.31	−0.03, 0.64	0.074	0.21	−0.07, 0.50	0.138
Albumin (g/dL)	0.01	−0.02, 0.04	0.530	0.01	−0.02, 0.05	0.414
Glucose (mg/dL)	0.22	−0.87, 1.30	0.695	−1.02	−5.33, 3.29	0.642
Total cholesterol (mg/dL)	−2.34	−5.74, 1.06	0.176	1.87	−1.44, 5.17	0.267
Triglyceride (mg/dL)	−1.27	−13.74, 11.19	0.840	−4.30	−11.48, 2.88	0.239
Potassium (mmol/L)	−0.04	−0.09, 0.01	0.075	−0.01	−0.05, 0.04	0.962
Total calcium (mg/dL)	0.04	−0.01, 0.09	0.067	−0.02	−0.07, 0.03	0.352
Phosphorus (mg/dL)	−0.08	−0.14, −0.02	0.014*	0.02	−0.04, 0.07	0.605
Ca × P (mg^2/dL^2)	−0.57	−1.19, 0.05	0.071	0.09	−0.47, 0.64	0.759

Multivariate linear regression analysis was conducted after adjusting for age; sex; hypertension; hyperlipidemia; eGFR; daily energy intake and protein intake; and use of statins, diuretics, sodium bicarbonate, potassium binders, and calcium carbonate.

The interaction between diabetic status and increased plant protein intake on serum phosphorus was statistically significant (*p* for interaction = 0.026*).

DM, diabetic mellitus; PRAL, potential renal acid load; HCO3, bicarbonate; Ca × P, calcium–phosphorus product.

*$p < 0.05$ was considered significant.
